# Targeted Overexpression of α-Synuclein by rAAV2/1 Vectors Induces Progressive Nigrostriatal Degeneration and Increases Vulnerability to MPTP in Mouse

**DOI:** 10.1371/journal.pone.0131281

**Published:** 2015-06-26

**Authors:** Lian-Kun Song, Kai-Li Ma, Yu-He Yuan, Zheng Mu, Xiu-Yun Song, Fei Niu, Ning Han, Nai-Hong Chen

**Affiliations:** 1 State Key Laboratory of Bioactive Substances and Functions of Natural Medicines, Institute of Materia Medica & Neuroscience Center, Chinese Academy of Medical Sciences and Peking Union Medical College, Beijing, 100050, China; 2 Hunan University of Chinese Medicine, Changsha, 410208, China; 3 Institute of Medical Biology, Chinese Academy of Medical Sciences and Peking Union Medical College, Kunming, China; Hertie Institute for Clinical Brain Research and German Center for Neurodegenerative Diseases, GERMANY

## Abstract

Mutations, duplication and triplication of *α-synuclein* genes are linked to familial Parkinson’s disease (PD), and aggregation of α-synuclein (α-syn) in Lewy bodies (LB) is involved in the pathogenesis of the disease. The targeted overexpression of α-syn in the substantia nigra (SN) mediated by viral vectors may provide a better alternative to recapitulate the neurodegenerative features of PD. Therefore, we overexpressed human wild-type α-syn using rAAV2/1 vectors in the bilateral SN of mouse and examined the effects for up to 12 weeks. Delivery of rAAV-2/1-α-syn caused significant nigrostriatal degeneration including appearance of dystrophic striatal neurites, loss of nigral dopaminergic (DA) neurons and dissolving nigral neuron bodies in a time-dependent manner. In addition, the α-syn overexpressed mice also developed significant deficits in motor function at 12 weeks when the loss of DA neurons exceeded a threshold of 50%. To investigate the sensitivity to neurotoxins in mice overexpressing α-syn, we performed an MPTP treatment with the subacute regimen 8 weeks after rAAV injection. The impact of the combined genetic and environmental insults on DA neuronal loss, striatal dopamine depletion, dopamine turnover and motor dysfunction was markedly greater than that of either alone. Moreover, we observed increased phosphorylation (S129), accumulation and nuclear distribution of α-syn after the combined insults. In summary, these results reveal that the overexpressed α-syn induces progressive nigrostriatal degeneration and increases the susceptibility of DA neurons to MPTP. Therefore, the targeted overexpression of α-syn and the combination with environmental toxins may provide valuable models for understanding PD pathogenesis and developing related therapies.

## Introduction

Parkinson’s disease (PD) is an age-related and the second most common neurodegenerative disorder. Clinical manifestations include motor impairments involving bradykinesia, resting tremor, rigidity and postural instability. Progressive loss of dopaminergic (DA) neurons in the substantia nigra (SN) and formation of Lewy Bodies (LB), which are cytoplasmic inclusions mainly containing *α*-synuclein (*α*-syn), are the pathological hallmarks of PD [1]. Duplication, triplication and mutations of *α-synuclein* genes are implicated in familial early-onset PD patients [2, 3] and genome-wide association studies also suggest a critical linkage between *α*-syn and PD pathogenesis [4, 5]. Studies have also implicated that the overexpressed wild-type, mutated or truncated *α*-syn contributes to cell toxicity *in vitro* and *in vivo*, which further suggests that elevated *α*-syn is involved in the pathogenesis of PD [6–9].

Although transgenic mice overexpressing wild type or mutated forms of *α*-syn have been produced, most of them have failed to replicate key pathological features such as the progressive loss of nigrostriatal DA neurons and neurites [10, 11]. With the exception of different forms and expression levels of *α*-syn, it’s probable that some compensatory mechanisms during development have counteracted the toxic effects caused by transgenes in these models. Therefore, conditional knockouts or viral vector mediated delivery of related genes may be an alternative approach to generate animal models that closely reproduce PD-like neurodegeneration[12]. To date, adeno-associated virus (AAV) and lentivirus have been used to overexpress *α*-syn in rats, and these models have replicated many PD-like pathological features [13–16]. However, the viral vector generated models show variability in neurodegeneration degrees and time frames [6, 13, 14, 17–20].

Most cases of PD are sporadic, which suggests a linkage between environmental factors and the pathogenesis. Exposure to pesticides such as rotenone, paraquat and maneb correlates with an increased incidence for PD [21], thus these agents have been used to generate PD-like animal models [22, 23]. The neurotoxin 1-methyl-4-phenyl-1,2,3,6-tetrahydropyridine (MPTP) has been discovered to induce human parkinsonism [24, 25], since then it has been used for modeling parkinsonism in rodents and non-human primates [26, 27]. Genetic susceptibility and exposure to environmental toxins probably contribute to the pathogenesis in combination.

In the present study, we used the rAAV2/1 vector to overexpress human wild type *α*-syn in the bilateral SN of mice. Since PD affects the brain bilaterally, bilateral lesions mimic the real pathological situations and behavior tests used for bilateral lesions are usually easy to perform. The overexpression of *α*-syn led to a progressive loss of nigral DA neurons and striatal TH positive neurites accompanied by motor behavior deficits during a period of 12 weeks. Meanwhile, the neurotoxin MPTP was administered to investigate the sensitivity of mice overexpressing *α*-syn. We observed a significant decrease in striatal dopamine, and a substantial loss of DA neurons and neurites after MPTP treatment. The evaluation of DA neuron survival, striatal tyrosine hydroxylase expression, dopamine levels, and phosphorylated and accumulated forms of *α*-syn revealed an increased vulnerability to the neurotoxin MPTP in the *α*-syn overexpression mice.

## Materials and Methods

### Recombinant adeno-associated virus 2/1 preparation and cell culture

The production of recombinant adeno-associated virus 2/1 (rAAV2/1) vectors were previously described [28]. Briefly, a human *α*-syn gene which was obtained previously [29] or GFP was inserted into the transfer plasmid to construct pSNAV- *α*-syn or pSNAV-EGFP, which encoded *α*-syn or EGFP transgene under the control of the CMV promoter. The transfer plasmids were transfected into BHK-21 cells (ATCC) using Lipofectamine 2000 (Invitrogen), then the confluent cells were infected by helper virus HSV1-rc/△UL2, which was essential for viron packaging. Cultures were collected until all of the cells were easy to fall into medium by vigorous shaking. The rAAV2/1- *α*-syn and rAAV2/1-EGFP vectors were purified by chloroform and NaCl. Chloroform (10%, v/v) was added to the collected cells, which were incubated at 37°C with vigorously shaking until all cells were lysed. Then solid NaCl was added till the final concentration was 1 mol/L by shaking at room temperature. The supernatant was harvested and PEG 8000 was added and centrifuged, then discarded the supernatant. Re-suspend the pellets in PBS buffer and add DAase I and RNase to obtain the final concentration of 1 μg/mL, then the culture was incubated for 30 minutes. An equal volume of chloroform was added to the suspension and the organic aqueous phases were separated by centrifugation. The aqueous phases containing the rAAV vectors were collected and referred to as the purified stock. Then dot blot hybridization was used to determine the genome particles of virus. The final titer for both vectors were 3.1×10^11^ genome copies/ml, aliquoted viruses were stored at -80°C and kept on ice during surgery.

HEK293 cells (ATCC) were maintained in Dulbecco’s modified Eagle’s medium (DMEM, Invitrogen) supplemented with 10% fetal bovine serum (FBS, Invitrogen), 100 unit/ml penicillin, 100 μg/ml streptomycin and 2 mM L-glutamine. Cells were maintained at 37°C with 5% CO_2_.

### Animals and surgical procedures

Adult male C57BL/6 mice weighing 18–22g were used for surgery and housed four to five per cage with access to food and water during a 12h light/dark cycle. Before surgery, mice were anesthetized with chloral hydrate. Then they were placed in a stereotaxic flame and the rAAV vectors were injected into the SN using the 10μl Hamilton syringe. 2μl rAAV2/1-α-syn solutions were infused bilaterally at a rate of 0.2μl/min and the needle was left for an additional 10mins before it was retracted. The same volume of rAAV2/1-EGFP vectors were infused bilaterally as the control group. All injections were made into the substantia nigra at stereotaxic coordinates: AP -3.2mm, ML 1.4mm, and DV -4.5mm below dura.

Mice injected with rAAV2/1-EGFP and rAAV2/1-α-syn vectors were used to investigate the sensitivity to neurotoxin MPTP. These mice were treated with 30mg/kg free base MPTP or saline for 5 consecutive days (i.p.) after 8 weeks of vector infusion and the animals were sacrificed 2 weeks after the last MPTP injection. All procedures were performed in accordance with the guidelines of National Institutes of Health for the care and use of laboratory animals and animal study was approved by the Animal Care Committee of the Peking Union Medical College and Chinese Academy of Medical Sciences. All efforts were made to minimize suffering.

### Behavioral testing

#### Open-field test

Locomotion activity was assessed in the open field apparatus at 4, 8, 12 weeks after vectors injection. A white plastic box (50×50×30 cm) was used and the area was divided into 25 grids of 100cm^2^. Mouse was placed individually in the center of the box and their behavior was video-taped, the following behavioral parameters were measured manually during the subsequent 5 min in normal lighting: horizontal locomotion (number of grids crossed) and the rearing frequencies (rearing activity). The equipment was cleaned with 70% alcohol and water between trials to avoid olfactory issues.

#### Swim test

Swim test was carried out in plastic containers [30]. The depth of water was 12cm and the temperature was maintained 22–25°C. Each mouse was scored in 1-min for 3 times with an interval of 10 mins. The score was a modification of that used by Marshall and Berrios: continuous swimming, 3; occasional floating, 2.5; floating time >50% of testing, 2; occasional swimming, 1.5; occasional swimming using hind limbs while floating on one side, 1; hind part sinks with head floating, 0. The observer was blind to the tested mice.

#### Pole test

A wood pole which was 50cm long and 1cm in diameter was used in pole test. It was wrapped in gauze to avoid slipping and mouse was put on the top ball of the pole. The time the mouse turned its nose down (inversion time) and the total time to climb down the pole were recorded. The mouse was guided if it didn’t move in 1min. Mice were all pre-trained before surgery, and during test each mouse performed 3 trials with an interval of 5 min. Three trials were averaged for statistical analyses.

### Immunochemistry and immunofluorescence

Mice were anesthetized using chloral hydrate (400mg/kg, i.p.) and then perfused with 0.1M phosphate buffered saline (PBS), followed by 4% paraformaldehyde and 4% paraformaldehyde with 3% w/v sucrose. Then the brain was moved and post-fixed in 20% sucrose (20% w/v in 4% paraformaldehyde) overnight. The brains were changed into 40% sucrose solution (40% w/v in 4% paraformaldehyde) after sinking.

Brains were sectioned on a freezing microtome (Leica) at thickness of 35μm. Floating sections were quenched with 3% hydrogen peroxide for 10 min followed by three washes of PBS, then incubated with 3% normal goat serum blocking for one hour. After that, the sections were incubated with antibodies overnight at 4°C. The antibodies were against tyrosine hydroxylase (TH) (1:200, sc-14007, Santa Cruz, Dallas, TX) or GFP (1:500, G6539, Sigma, St. Louis, MO), human α-syn (1:500, Syn204, Cell Signaling Technology, Danvers, MA), mice and human α-syn (1:200, sc-7011-R, Santa Cruz), phospho- α-syn (1:200, phosphor-S129, ab51253, Abcam, Cambridge, MA) and Neuronal Nuclei (NeuN) (1:500, MAB377, Millipore, Temecula, CA). Sections were rinsed three times in 0.1% tween 20-PBS and then incubated with HRP labeled secondary antibodies (1:200) 2 hours at room temperature. After washes, sections were visualized using 3, 3-diaminobenzidine (DAB) and coverslipped.

Fluorescence immunostaining was performed as above without quenching, the Alexa488 or 546 conjugated antibodies (Invitrogen, 1:200, Carlsbad, CA, USA) were used in the dark. The slides were examined using a Zeiss laser scanning confocal microscope (LSCM).

### Unbiased stereology

Numbers of TH-positive neurons in substantia nigra pars compacta (SNpc) were estimated using Stereo Investigator (MBF Bioscience, USA) as described[31]. We used one in six sections to assess the number of TH neurons in SNpc of both hemisphere and the counts were made automatically within a counting frame of 100μm×100μm area. A systematic random sample area was created by the Stereo Investigator which randomly positioned the counting frame within the SNpc under a magnification of 40×. The z-dimension of the counting brick was defined 16μm and a 2μm guard was used. Cells were counted within TH staining outlines and the total estimates were obtained. Density of striatal TH-positive fibers was measured by densitometry using Image-pro plus software 6.0. The values were corrected for non-specific background staining values from cortex. The rAAV-GFP injected mice were used as control.

### Western Blotting

Mice were sacrificed at 4, 8 and 12 weeks after vector injection and 2 weeks after the last MPTP treatment, brains were removed rapidly and midbrain and bilateral striatum were dissected quickly then snap-frozen on dry ice. Tissues were homogenized in RIPA lysis buffer (with protease inhibitor cocktail, phosphatase inhibitors and PMSF). Homogenates were centrifuged at 4°C and the supernatants were stored at -80°C. Protein concentration was determined by bicinchoninic acid protein assay and proteins were boiled for 5 min in loading buffer. A total of 20μg proteins were separated by SDS-PAGE and then transferred to PVDF membrane (Millipore). After blocking with 3% BSA 2 hours, membranes were incubated with the following primary antibodies: anti-TH (1:500, Santa Cruz, Dallas, TX), anti-dopamine transporter (DAT) (1:500, Santa Cruz, Dallas, TX), anti-human α-syn (1:500, CST, Danvers, MA) overnight at 4°C. After washing in TBST (Tris-buffered saline with 0.1% tween-20) with gentle agitation, membranes were incubated with HRP-conjugated secondary antibodies for 2 hours at room temperature. The bands were detected using enhanced chemiluminescence (GE). Densitometric analysis of each protein was conducted using Gel-pro analyzer software (Media Cybernetics).

### Nissl staining

Brain sections were stained with cresyl violet and washed in distilled water, then dehydrated through graded alcohols (70%, 95% and 100%), placed in xylene and coverslipped. The counting frame of 100μm×100μm area was used within the SNpc under a magnification of 40μ and normal neuronal bodies were identified by size. One in six sections was used for neuronal counting and the analysis was performed on six mice of each group. The rAAV-GFP transduced mice of 12 weeks were used as control.

### HPLC

Dopamine and metabolites were detected by high-performance liquid chromatography with electrochemical detection (Waters 2465 electrochemical detector). Mice were sacrificed and striatal tissues were dissected and quickly frozen in liquid N_2_. Samples were homogenized in 0.1M perchloric acid. After centrifugation, the supernatant was filtered and 20μl of it was injected into Waters e2685 separations module equipped with a Diamonsil (5μ,100A) C18 HPLC column (150×4.60mm) as previously described [21, 32]. The mobile phase of HPLC contains 85mM citric acid, 100mM anhydrous sodium acetate, 0.2mM EDTA, 0.5mM octane-1-sulfonic acid, 15% (v/v) methanol in distilled water, pH3.68, and the flow rate was 1.0mL/min. A standard curve generated from injection of standards of highest purity was used for the quantification.

### Statistics

Statistics were analyzed by GraphPad Prism 5.0 and SPSS 17.0 Software. A two-way analysis of variance (ANOVA) was performed for the time-dependent treatment, differences among group means were analyzed using Bonferroni *post-hoc* test. Swim test was analyzed using Wilcoxon signed ranks test by SPSS. Other experiments were analyzed using Student’s t-test or one-way ANOVA followed by a Newman-Keuls *post-hoc* test. All values are presented as mean±SEM. Statistical significance was set at *P*<0.05.

## Results

### Efficient overexpression of α-syn or GFP after delivery of rAAV vectors

**Fig 1:**
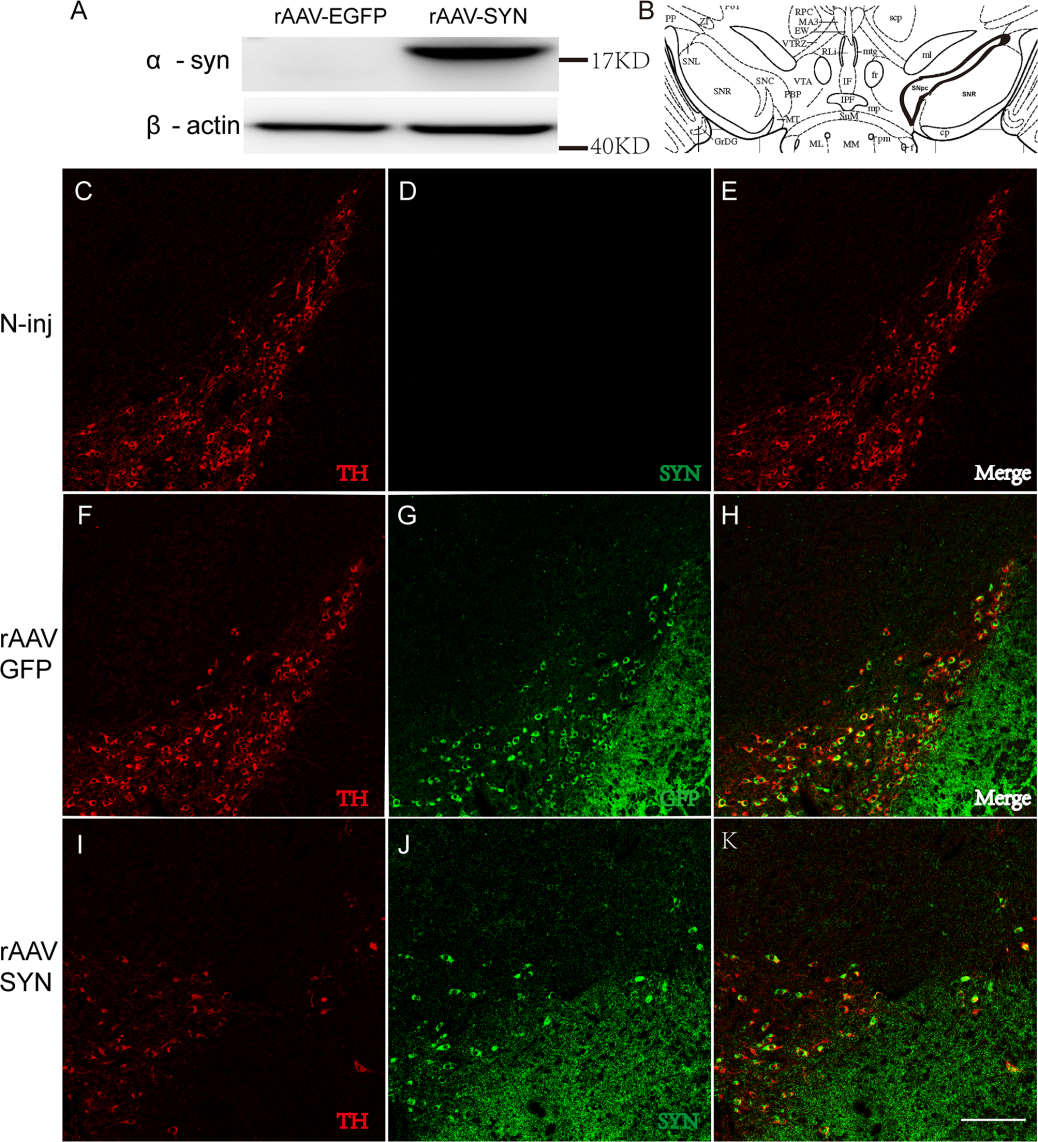
Overexpression of α-syn in the SNpc after rAAV-α-syn transduction. Western blotting showed a significant expression of human-**α**-syn in HEK293 cells 96 hours after transduction (A). The position of SNpc was indicated (B) and TH positive staining (Red) indicated a loss of DA neurons in the SNpc 8 weeks after rAAV-**α**-syn injection, however, the co-staining of human-**α**syn (Green) was also observed in the existing DA neurons (I-K). The overexpression of GFP was also detected in TH-positive neurons in the SNpc after rAAV2/1-GFP injection (F-H). And no α-syn staining was observed in the none-injected mouse (C-E). TH staining confirmed no loss of DA neurons in GFP and none injected group. Antibody against human α-syn was used (CST). Scale bar: 100μm.

rAAV2/1-**α**-syn vectors were firstly transduced to HEK293 cells to verify the overexpression of **α**-syn, and rAAV2/1-EGFP vectors were used as the negative control. We analyzed total cell lysates by western blotting and the result revealed that full length human **α**-syn was highly expressed in rAAV2/1-**α**-syn vectors transduced cells but not in cells transduced by rAAV2/1-EGFP vectors (Fig 1A).

After that, we investigated the effect of overexpressed α-syn in SN at 4 (S1 Fig) and 8 weeks. The expression of α-syn or GFP were evaluated by performing double immunofluorescence staining. Co-immunoreactive staining of human α-syn and TH showed a significant loss of TH-positive neurons in the SNpc, but we also observed an efficient overexpression in the surviving nigral neurons of rAAV2/1-α-syn infected mice (Fig 1I, 1J and 1K). In contrast, neither human α-syn positive staining nor loss of TH positive neurons appeared in the SN of uninjected mice (Fig 1C, 1D and 1E). And we observed a high expression ofGFP with no insult in the SN of rAAV2/1-EGFP infected mice (Fig 1F, 1G and 1H). These results demonstrated an efficient transduction of α-syn or GFP in nigral DA neurons.

### The overexpression of α-syn induces progressive loss of nigral neurons, striatal neurites and morphological changes of neurons in SNpc

**Fig 2:**
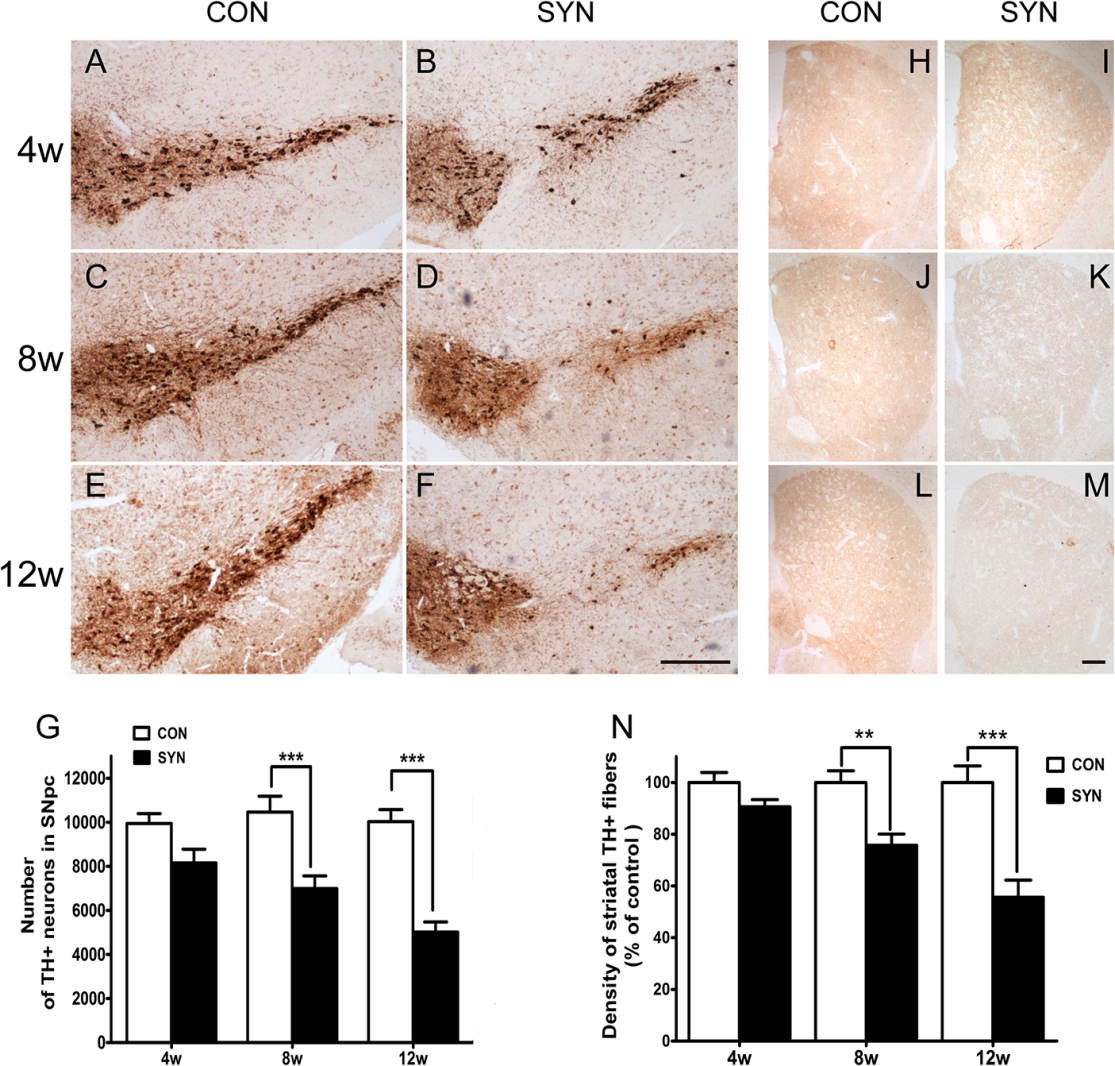
Immunohistological analysis of DA neurons in the SNpc and TH positive neurites in the striatum. Representative micrographs of nigral sections immunostained for TH in mice at 4, 8 and 12 weeks after delivery of rAAV-GFP (CON: A, C and E) and rAAV-α-syn (SYN: B, D and F) vectors. Scale bar: 400μm. Illustration of striatal TH positive fiber density over time (H, J, L: rAAV-GFP; I, K, M: rAAV-α-syn). Scale bar: 50μm. Quantification of TH positive neurons in the SNpc and striatal TH immunoreactive fiber density was shown (G, N). Data are means ± SEM of 6 mice, **P<0.01, ***P<0.001 as compared to rAAV-GFP control (2-way ANOVA, Bonferroni *post hoc* test).

**Fig 3:**
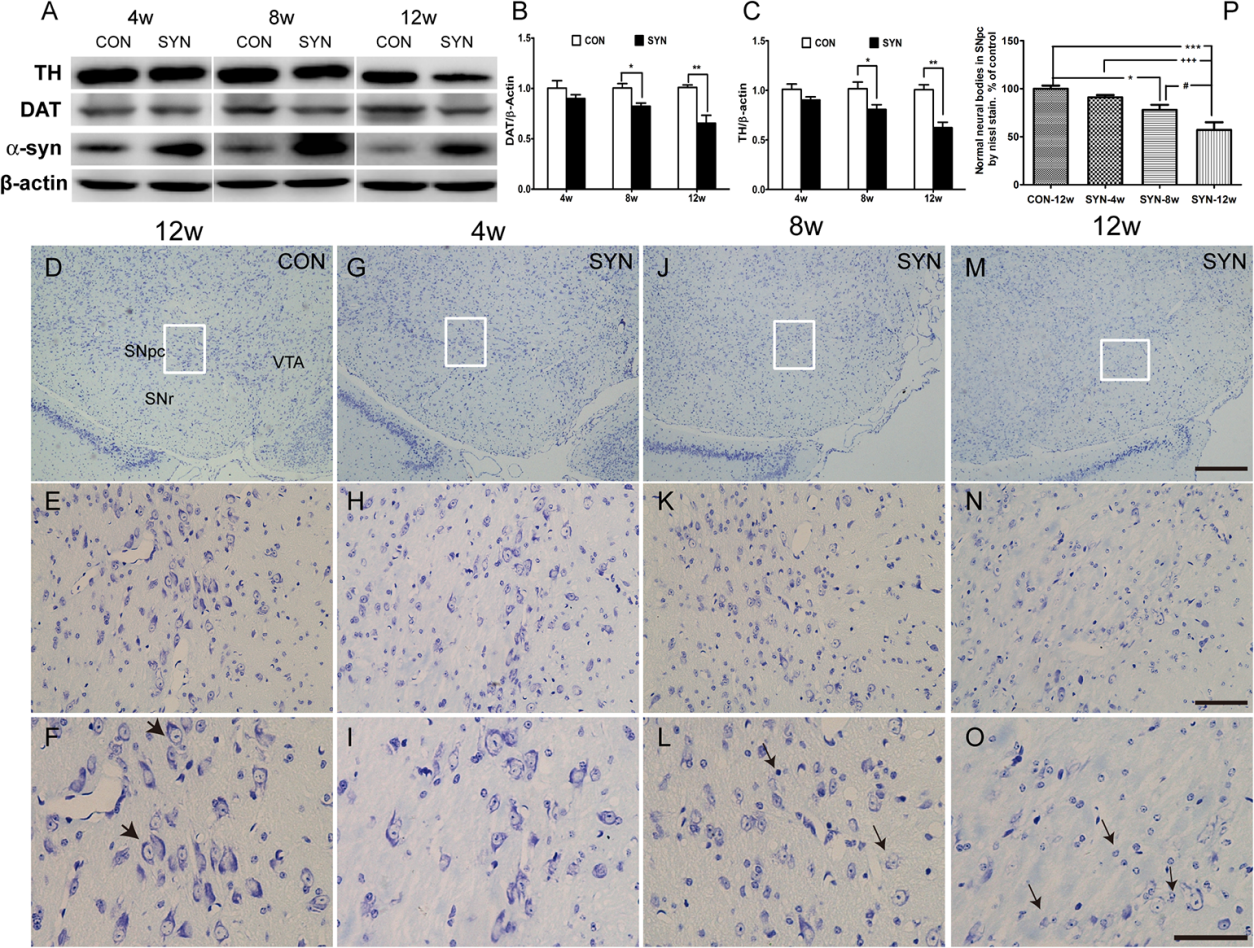
Protein levels of α-syn, TH and DAT in midbrain and morphology of DA neurons in the SNpc. Representative bands for TH, DAT, α-syn and β-actin in the midbrain at 4, 8 and 12 weeks of the rAAV-GFP (CON) and rAAV-α-syn (SYN) transduced mice (A). The densities were analyzed as protein/β-actin ratio and normalized by the results of CON. Data are presented as mean±SEM of 3 mice (B, C), *P<0.05, **P<0.01 compared with the GFP control (unpaired, two-tailed Student’s *t* test). (Antibody against α-syn: Santa Cruz, sc-7011-R). Neuronal morphology exhibited by nissl stain at 4 (G-I), 8 (G-L) and 12 weeks (M-O) after rAAV-α-syn injection. Note that bigger arrowheads indicate normal staining of nissl bodies in nigral neurons at 12 weeks of rAAV-GFP transduced mice (F) and the smaller arrows denote dissolving nissl bodies of neurons. Numbers of normal staining neuronal bodies in the SNpc were shown as percentage of the numbers in GFP control mice (P). Data are means ± SEM of 6 mice, #, *P<0.05, +++, ***P<0.001 (one-way ANOVA Newman-Keuls *post-hoc* test). Scale bars: 200μm (D, G, J, M); 50μm (E, H, K, N); 50μm (F, I, L, O).

Mice were sacrificed for histological analysis at 4, 8, and 12 weeks after delivery of rAAV2/1-α-syn or rAAV2/1-EGFP vectors. Immunohistochemical staining for TH revealed that the overexpression of α-syn induced a progressive loss of dopaminergic neurons in the SNpc compared with the GFP injected control (Fig 2A, 2B, 2C, 2D, 2E and 2F). And the quantifications indicated a significant loss of TH+ neurons in SNpc at 8 weeks (34% reduction; *P*<0.001 compared to control) and the deficit was more pronounced at 12 weeks (50% reduction, *P*<0.001) (Fig 2G). Furthermore, protein levels of TH and dopamine transporter (DAT) in the midbrain also largely depleted at 8 and 12 weeks after rAAV-α-syn transduction (Fig 3A). We also observed more dissolving and disappearing nissl bodies in neurons of SNpc 12 weeks after α-syn overexpression (Fig 3M, 3N and 3O) and cell counting showed significant decrease of normal neuronal bodies in the SNpc of α-syn overexpressing mice (Fig 3P), which demonstrating a progressively developed insult to nigral neurons.

To evaluate the impact of α-syn on axonal terminals of nigral DA neurons, optical density of striatal TH-positive fibers was assessed (Fig 2H, 2I, 2J, 2K, 2L and 2M). Similar to the results observed in neurons of the SNpc, TH staining density of striatal fibers reduced gradually over time. Loss of striatal TH positive fibers was observed ranging from 10% at 4 weeks to 25% at 8 weeks (*P*<0.01) and a marked reduction of 45% at 12 weeks(*P*<0.001) (Fig 2N).

### Impaired motor behavior induced by α-syn overexpression

**Fig 4:**
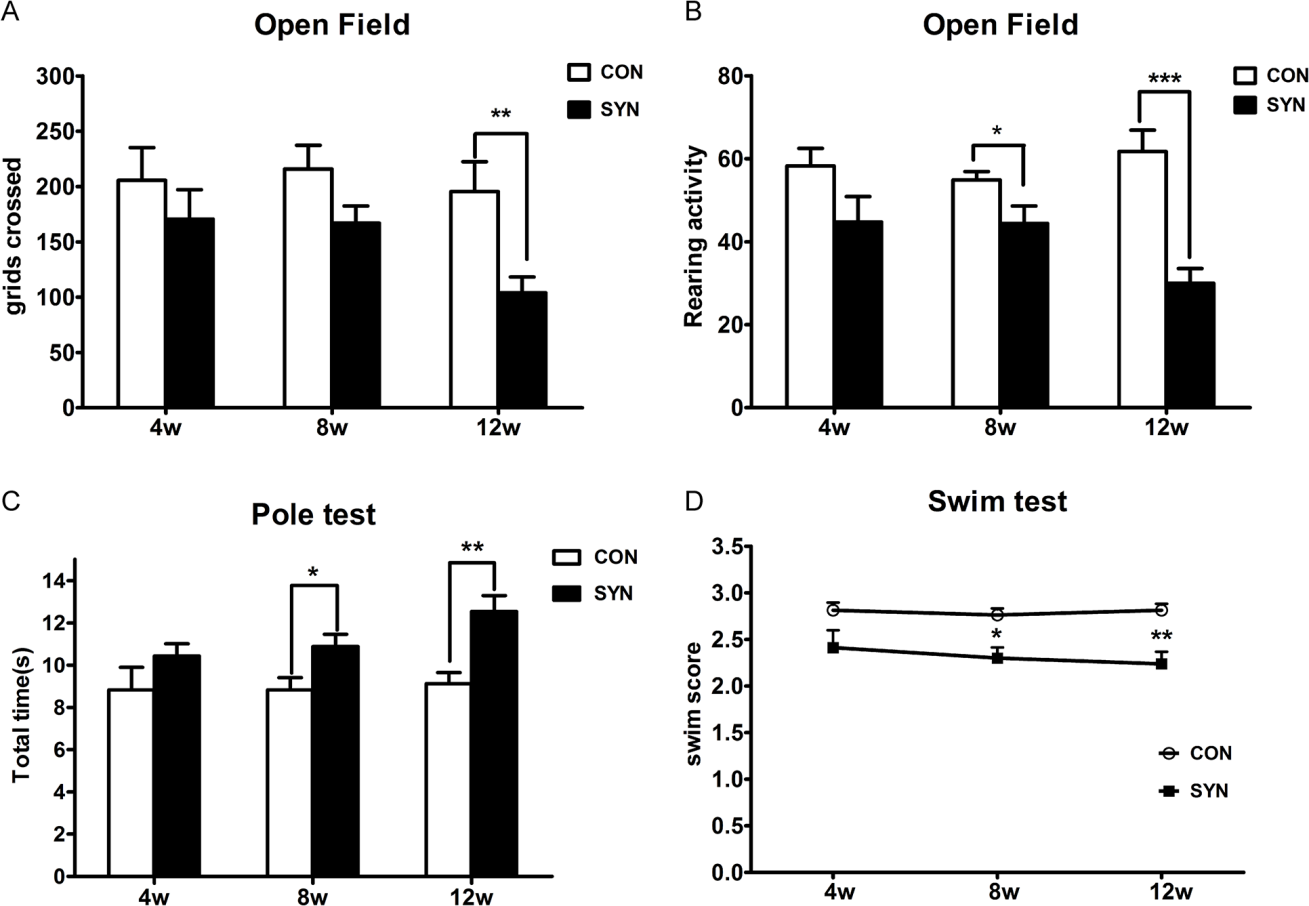
Motor behavior assessment. Motor functions were assessed in open-field arena, pole test and swim test. Spontaneous horizontal locomotor activities (A) and rearing activities (B) at 4, 8, and 12 weeks after rAAV-α-syn injection (SYN), in comparison with rAAV-GFP group (CON). Total time to descend the pole (C) and the behavioral score (D) in swimming test of rAAV-α-syn transduced mice, in comparison with rAAV-GFP mice. Data are expressed as meanαSEM of 8 mice. *P<0.05, ** *P*<0.01, ***P<0.001 (unpaired, two-tailed Student’s *t* test for open-field and pole test; Wilcoxon signed rank tests for swimming test).

We performed varieties of behavioral tests to evaluate the functional effects of overexpressing α-syn or GFP in nigral neurons, and the mice were tested at 4, 8 and 12 weeks after injection. We found that mice overexpressing α-syn showed deficient locomotor activities compared to control group at 4 (Fig 4A, 170±26.7 vs. 205±29.5 squares) and 8 weeks (164±17.3 vs. 212±30.3 squares) in open field test, moreover, a significant decrease was observed at 12 weeks (104±14.2 vs. 195±26.9, *P*<0.01). In addition, the rearing activities in open field test also decreased significantly at 12 weeks (Fig 4B, 30±3.6 vs. 62±5.1, *P*<0.001). The locomotor deficiency was compatible with a loss of 50% DA neurons at 12 weeks.

Pole test is often used in MPTP treated rodents models to indicate bradykinesia. Mice in the α-syn group showed a delayed time to descend the pole and a lower score of the behavior on the pole (data not shown). The total time to climb down the pole increased significantly at 12 weeks (Fig 4C, 12.2±0.78s compared to 9.1±0.52s, *P<*0.01). Furthermore, scores in swim test (Fig 4D) also reduced significantly at 8 (*P*<0.05) and 12 weeks(*P*<0.01). These data demonstrated that progressive loss of DA neurons triggered by α-syn overexpression impaired the motor behavior function.

### Combination of MPTP and overexpressed α-syn leads to more loss of DA neurons and decline in motor function

**Fig 5:**
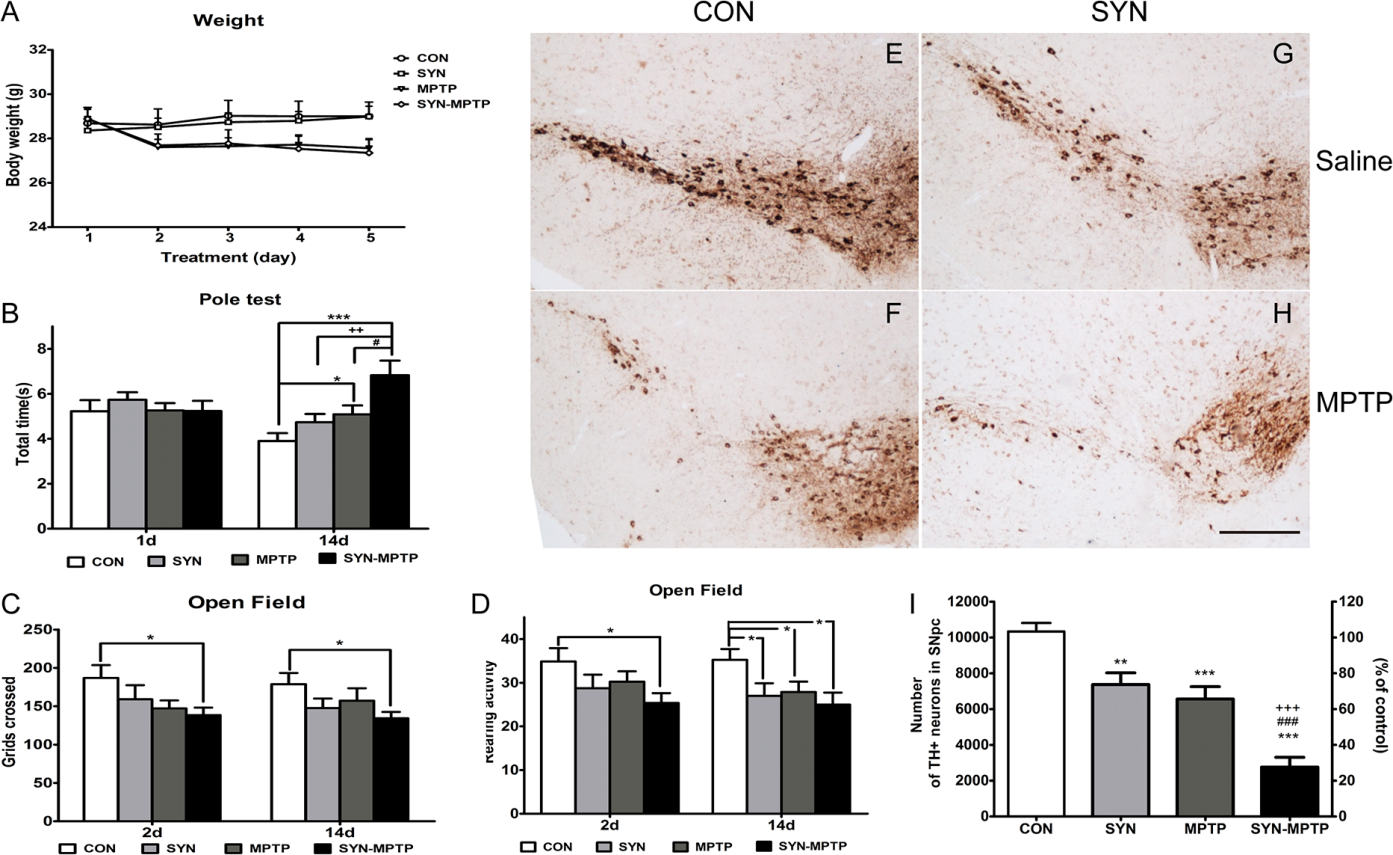
Motor dysfunction assessment and numbers of TH positive neurons in the SNpc after MPTP treatment. Body weights of mice during MPTP injection (5 consecutive days: A). *, compared to CON (rAAV-GFP+saline); +, compared to SYN (rAAV-α-syn+saline); #, compared to MPTP (rAAV-GFP+MPTP); SYN-MPTP (rAAV-α-syn+MPTP). ANOVAMotor dysfunction assessment: pole test was performed 1 and 14 days after the last MPTP injection (B), and locomotor activities as well as rearing activities in open field test were recorded 2 and 14 days after the last MPTP injection (C-D), data are means±SEM of 9 mice, *, +, #*P*<0.05, ++*P*<0.01, ***P<0.001 (one-way ANOVA Newman-Keuls *post-hoc* test). Representative images and quantification for TH positive neurons in the SNpc after MPTP treatment (E-I), scale bar: 400μm. Data are means±SEM of 6 mice, **P<0.01; ***, +++, ### *P*<0.001 (one-way ANOVA Newman-Keuls *post-hoc* test).

Abnormal α-syn was widely observed in neurotoxin induced PD models including the rodent MPTP model[33, 34], however, conflicting results on the sensitivity to MPTP were obtained in human α-syn overexpressing mice[35, 36]. One of the possibilities associated with the contradiction results may be the different patterns among α-syn overexpression models. To find whether α-syn overexpression mediated by rAAV vectors could make nigrostriatal system more susceptible to environmental neurotoxin, we treated the mice MPTP in a subacute regimen at 8 weeks after rAAV-α-syn infusion, when modest decline of nigrostriatal system was observed. Then behavioral activities were assessed after injections of 5 consecutive days (once per day), and during which time the body weight of mice treated with MPTP decreased slightly (Fig 5A). Twenty-four hours after the last MPTP injection, mice in α-syn-MPTP group didn’t exhibit significantly increased time to descend the pole compared to the other three groups. While on the 14^th^ day after the last MPTP injection, mice of α-syn-MPTP showed a declined motor behavior in pole test and the time to descend the pole significantly increased compared to mice in control (6.83±0.66 vs. 3.93±0.35s, *P*<0.001), α-syn (6.83±0.66 vs. 4.74±0.37, *P*<0.01), and control-MPTP (6.83±0.66 vs. 5.09±0.40, *P*<0.05) groups (Fig 5B). Locomotor activities in open-field test reduced 2 and 14 days after the last MPTP injection, rearing activities showed a notable decrease too (Fig 5C and 5D). Behavior tests suggested increased motor impairments to MPTP after the overexpression of α-syn.

**Fig 6:**
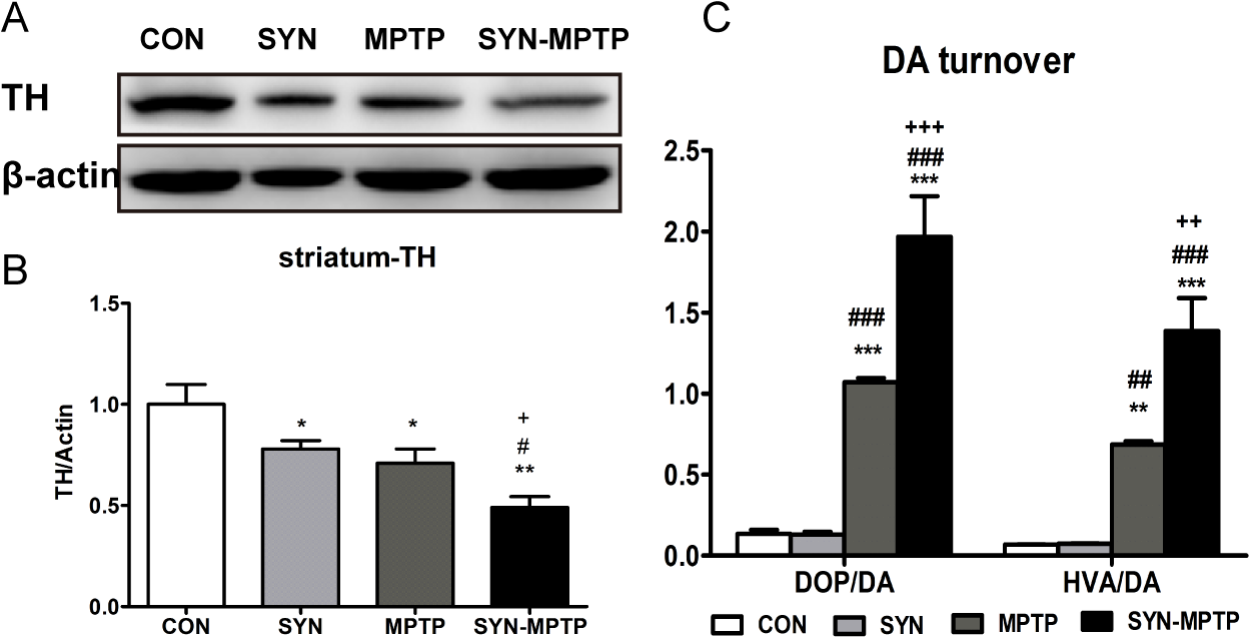
Loss of striatal protein TH and increased dopamine turnover after MPTP treatment. Protein blots for striatal TH were analyzed TH/β-actin ratio and normalized by the results of CON (A, B). Dopamine turnover, which was analyzed by DOPAC/DA and HVA/DA ratio, exhibited significant increase after MPTP treatment (C). Data are presented as mean±SEM of 3 mice; *, compared to CON; #, compared to SYN; +, compared to MPTP. *, +, #: *P*<0.05; **, ++, ##: *P*<0.01; ***, ###, +++: *P*<0.001 (one-way ANOVA Newman-Keuls *post-hoc* test).

To evaluate the pathological alterations of MPTP and α-syn to nigrostriatal system, we assessed the TH positive neurons in SNpc after MPTP treatment. We found more loss of TH positive neurons in the SNpc of α-syn-MPTP group compared with the α-syn group (Fig 5E, 5F, 5G, 5H and 5I) (75% reduction vs. 29% reduction, Newman-Keuls *post-hoc* test, *P*<0.001) and control-MPTP group (75% reduction vs. 37% reduction, *P*<0.001). Consistent with lesions of nigral DA neurons, a similar reduction of TH protein in striatum of α-syn-MPTP mice was also detected by western blotting (Fig 6A and 6B).

### Biochemical impairments after MPTP treatment

To determine the impact of MPTP on dopamine synthesis, storage and turnover, striatal dopamine (DA) and its metabolites, 4-dihydroxyphenylacetic acid (DOPAC) and homovanillic acid (HVA), and 5-hydroxytryptamin (5-HT) were assessed by HPLC (Table 1). Consistent with the motor impairment, a reduction of striatal DA level in MPTP mice was found (1.60±0.12 vs. 12.22±0.89 ng/mg, *P*<0.001), and the depletion was more pronounced in the α-syn-MPTP group (0.87±0.12 vs. 12.22±0.89ng/mg, *P*<0.001). Additionally, we found a significant increase in DA turnover, as measured by DOPAC/DA and HVA/DA ratios (Fig 6C). Ratio of DOPAC/DA exhibited an increase of 14 times in mice of α-syn-MPTP group compared to control, while the ratio of mice in MPTP group just increased approximately 8 times. And the HVA/DA ratio revealed a similar change to the DOPAC/DA ratio. The notable changes revealed a more pronounced inhibitory effect on neurotransmitters triggered by combination of MPTP and α-syn.

**Table 1:** Striatal dopamine depletion after MPTP treatment.

	Striatal level, ng/mg of tissue (mean±SEM)			
Treatment	Dopamine	DOPAC	HVA	5-HT
**CON**	12.22±0.89	1.69±0.40	0.85±0.09	0.41±0.07
**SYN**	9.31±0.86^*^	1.24±0.24	0.69±0.07^*^	0.43±0.08
**MPTP**	1.60±0.12^***^ ^,^ ^###^	1.71±0.01	1.09±0.00^##^	0.41±0.01
**SYN-MPTP**	0.87±0.12^***^ ^,^ ^###^	1.65±0.11	1.15±0.07^*^ ^,^ ^##^	0.31±0.12

*: *P*<0.05,

***: *P*<0.001 compared to CON;

##: *P*<0.01,

###: *P*<0.001 compared to SYN.

One-way ANOVA Newman-Keuls *post-hoc* test.

### Combination with MPTP induces more phosphorylation, accumulation and nuclear distribution of α-syn

**Fig 7:**
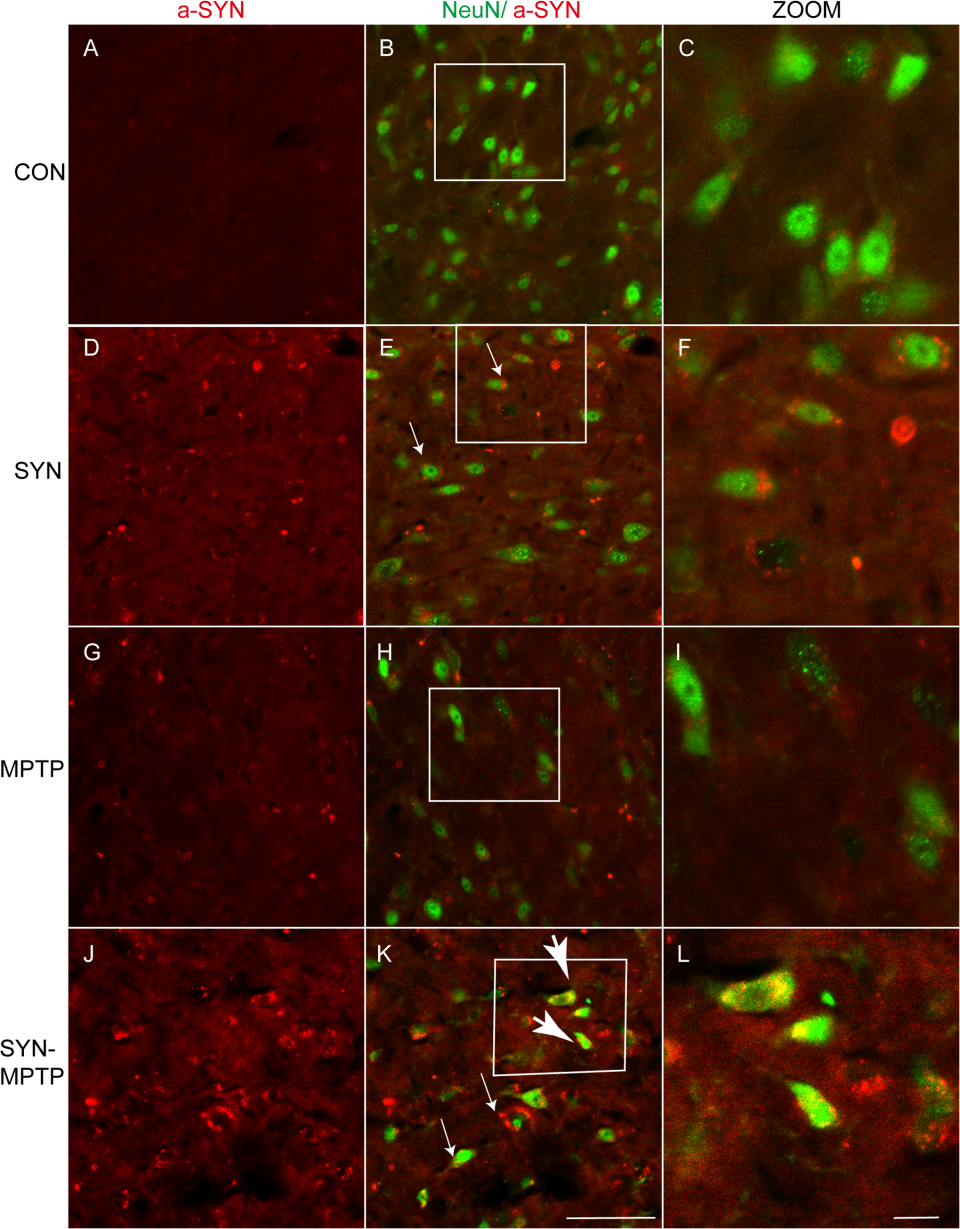
α-syn aggregation and nuclear distribution in nigral neurons after MPTP treatment. Double Immunofluorescence staining for NeuN (green) and **α**-syn (red) was performed to assess the condition of **α**-syn (antibody against **α**-syn, Santa Cruz, sc-7011-R). Small arrows denote dot-like accumulation of **α**-syn surround the nucleus after rAAV- **α**-syn transduction (E). More **α**-syn accumulation in cytoplasm (small arrows, K) and nucleus distribution (big arrow, K) in neurons of the **α**-syn and MPTP combined treatments. A-C: CON (rAAV-GFP+saline); D-F: SYN (rAAV- **α**-syn+saline); G-I: MPTP (rAAV-GFP+MPTP); J-L: SYN-MPTP (rAAV- **α**-syn+MPTP). Scale bar: 50um (A, B, D, E, G, H, J, K); 20μm (C, F, I, L).

Abnormal aggregation of **α**-syn is linked to its toxicity in animal models of PD and double fluorescence immunostaining was performed to visualize the condition of **α**-syn in DA neurons in SNpc. Though toxic forms of **α**-syn are not clearly confirmed, oligomeric, phosphorylated and other posttranslational modifications of **α**-syn may all contribute to lesions of DA neurons [37–39], and cell distribution of **α**-syn may also impact the neuronal toxicity. We found the overexpression of **α**-syn was maintained after MPTP treatment and the staining sizes increased surrounding the nucleus in **α**-syn transduced neurons (Fig 7D, 7E and 7F). Meanwhile, more **α**-syn positive accumulations were observed distributing in nucleus of neurons in the SNpc of **α**-syn+MPTP group (Fig 7J, 7K and 7L). Nuclear aggregation of **α**-syn may be toxic to neurons according to researches by Kontopoulos and Ma [40, 41]. Previous studies have shown that aggregation of **α**-syn can induce damage to mitochondria, autophagy, ubiquitin-protease system as well as production of ROS, thus destroy the normal function of neurons and contribute to neuronal degeneration [42, 43].

**Fig 8:**
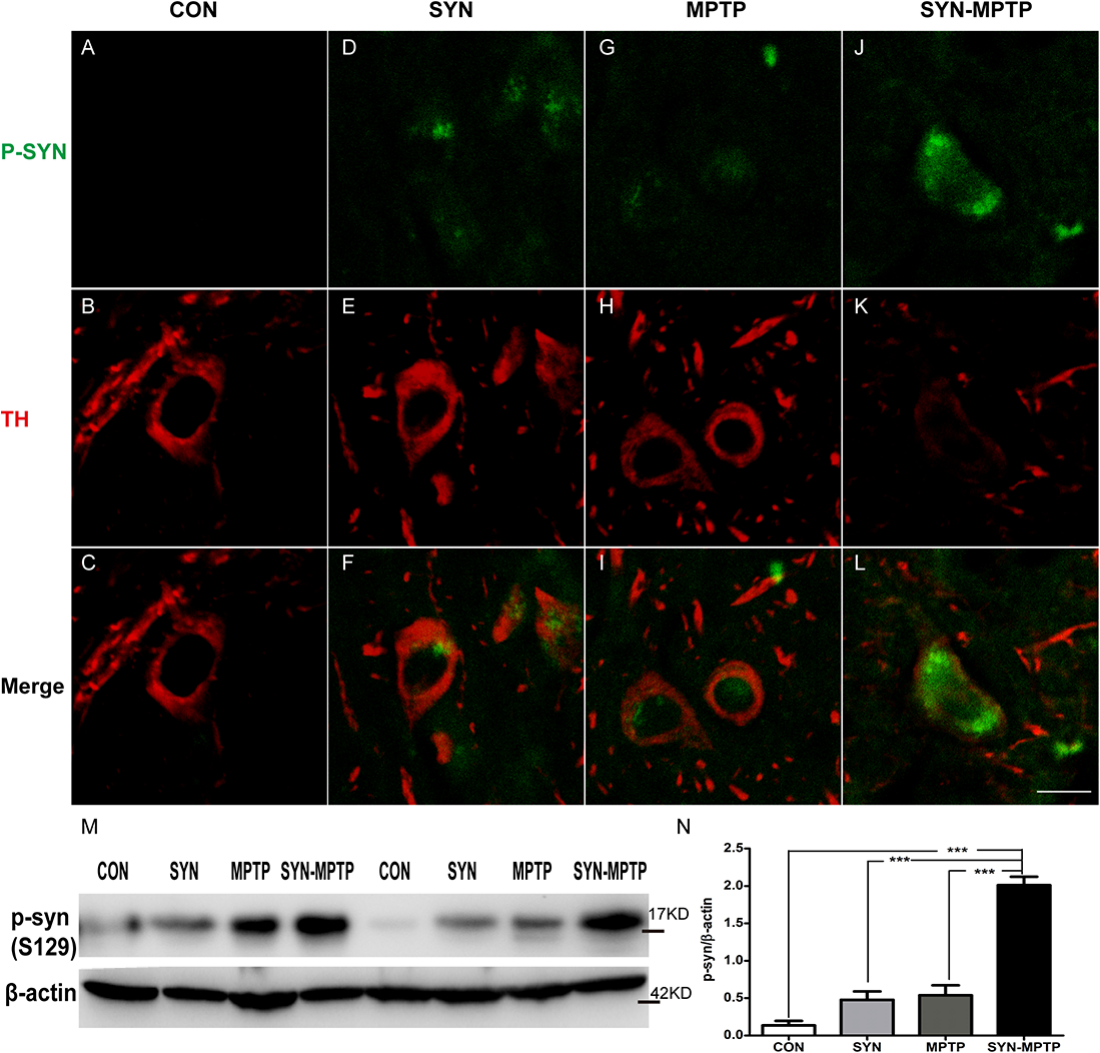
Accumulation of phosphorylated α-syn in TH positive neurons of SN and increased protein levels of phosphorylated α-syn in midbrain. Double Immunofluorescence staining for phosphorylated **α**-syn (P-S129) (green) and TH (red) was performed. Phosphorylated forms were observed in TH positive neurons of SYN (D-F) and MPTP (G-I) mice, furthermore, notable accumulation was also detected in DA neurons of the SYN-MPTP mice (J-L). Scale bar: 20μm. Phosphorylated **α**-syn (P-S129) was quantified by western blotting (M) and the accumulation in midbrain of SYN-MPTP mice was significant (N). Data are presented as mean±SEM of 3 mice; *P<0.05, ***P<0.001 compared to SYN-MPTP (one-way ANOVA Newman-Keuls *post-hoc* test).

After that, we visualized the phosphorylated forms in nigral DA neurons with an antibody specific for phosphor-Ser129- **α**-syn (Fig 8A, 8D, 8G and 8J). In **α**-syn+MPTP mice, we detected pronounced staining of phosphorylated forms distributing both in cytoplasm and nucleus of DA neurons, in which the TH staining was markedly decreased (Fig 8J, 8K and 8L). Moreover, phosphorylated **α**-syn was also detected in DA neurons of rAAV- **α**-syn and MPTP group (Fig 8D, 8E, 8F, 8G, 8H and 8I), while none was observed in DA neurons of control mice (Fig 8A, 8B and 8C). Consistent with the immunofluorescence staining, quantification for protein levels of the phosphorylated forms in midbrain of **α**-syn+MPTP mice also supported the significant accumulation (Fig 8M and 8N).

## Discussion

In the current study, we have induced widespread overexpression of human wild type **α**-syn in the mouse SN by stereotaxic delivery of rAAV2/1 vectors. A progressive lesion of DA neurons in the SNpc was observed after rAAV2/1- **α**-syn injection, and the loss of TH positive neurons in the SNpc increased from 30% at 8 weeks to nearly 50% at 12 weeks. Long term effects of the overexpressed **α**-syn consisted of degeneration of DA neurons in the SNpc, appearance of striatal dystrophic neurites throughout and motor dysfunction. Comparing to previous unilateral models, we performed a bilateral rAAV2/1- **α**-syn administration. The bilateral insults are similar to the situations in clinical patients and motor activities are easy detected in the behavioral experiments. Rodent models of PD by overexpressing **α**-syn in the nigrostriatal pathway using viral vectors are usually unilateral models. The overexpression of human **α**-syn in bilateral SN led to significant motor impairments and pathological damages in our study, however, most unilateral lesions by rAAV induced **α**-syn overexpression with comparative titers showed minimal neurodegeneration and behavioral impairments [6, 12, 14, 16, 17, 44]. The bilateral lesions may produce more damage than unilateral lesions and behavioral impairments may be more significant. In addition to bilateral lesions, different rAAV systems which could influence the transduction efficacy should also be considered.

Models based on rAAV mediated overexpression of human wild-type **α**-syn exhibit different timeframes and severity of pathology [6, 12, 14, 16, 17, 44]. The variability is most likely due to distinct serotypes of the rAAV virus as well as the different promoters and enhancers, which all lead to diverse transduction efficacy and selectivity of neurons. In addition, dose-dependent degeneration after rAAV- **α**-synuclein delivery has been implicated in research by Oliveras, which has demonstrated that the expression levels of **α**-syn notably influence the rates of neurodegeneration [17]. Moreover, rats, mice and non-human primates subjected to the same administration may display different time-courses of changes. It has been shown that the expression levels of **α**-syn should be considered as a primary factor in determining the extent and time-span of neurodegeneration.

In addition to triggering degeneration of DA neurons and neurites, the overexpression of **α**-syn led to dysfunction of motor activities monitored in spontaneous behavioral tests. Motor behavior impairments appeared to be significant at 12 weeks after injection of rAAV2/1- **α**-syn vectors. Previous studies have shown that only more than half loss of DA neurons or depletion of striatal DA was sufficient to induce significant behavioral manifestations. So in most instances, virus-mediated models of **α**-synuclein overexpression do not display significant behavioral deficits [45].

Etiology of PD is complex, both clinical and experimental studies have proposed that the interactions between genetic and environmental factors are involved in the disease. Neurotoxins such as 6-hydroxydopamine (6-OHDA), MPTP, rotenone and paraquat can mimic one or more characteristics of PD, thus they are widely used to create PD models. MPTP is converted to 1-methyl-4-phenylpyridium ion (MPP^+^) by monoamine oxidase B (MAO-B) in glia and serotonergic neurons after crossing the blood–brain barrier [46]. And MPP^+^ is released into extracellular space and selectively transported into neurons by DAT [47]. It inhibits function of mitochondria complex I and induces oxidative stress, thus to exert cytotoxic effects [48].

C57BL/6 mice are more sensitive to MPTP than rats, and three MPTP regimens have been used to induce nigrostriatal damage: acute, subacute and chronic [27]. MPTP models are not sufficient to produce progressive neurodegeneration, therefore nigrostriatal lesions are usually detected one or two days after the last MPTP injection, and partial recovery has been observed in acute and subacute regimens at longer time points after MPTP administration [49]. Therefore, we treated the rAAV- **α**-syn transduced mice with MPTP to investigate the impact of combined genetic and environmental insults.

MPTP administration after **α**-syn overexpression induced greater nigrostriatal impairments compared to the **α**-syn+saline and control+MPTP groups, and deficits in motor activities were observed in pole and open field test 14 days after the last injection. Moreover, a neurochemical analysis indicated markedly decreased striatal DA levels and increased DA turnover after MPTP administration. These results demonstrate an increased susceptibility of mice to MPTP after nigral **α**-syn overexpression. In addition, our findings suggest that the neurotoxin MPTP induces more striatal DA reduction than **α**-syn. Phosphorylation of **α**-syn at the serine residue in position 129, a post-transcriptional modification, is also considered to be associated with **α**-syn pathology [50–52]. Positive staining of S129-phosphorylated x-syn increased significantly after the combined treatment of **α**-syn and MPTP. Some *in vitro* and *in vivo* studies have suggested that the phosphorylated form of **α**-syn is toxic to DA neurons [53–55].

The overexpression of **α**-syn can induce increased oxidative stress and decreased mitochondria complex I activity. The oxidative stress in turn may induce **α**-syn to aggregate into toxic forms, which would further damage mitochondria and produce more ROS [56, 57]. Therefore, the overexpressed **α**-syn probably leads to impaired mitochondria more vulnerable to MPTP and induces more insults. Furthermore, the overexpressed **α**-syn is able to impact the protein degradation system including the ubiquitin-proteasome system and autophagy [58, 59]. The damaged protein degradation system in turn leads to more **α**-syn accumulation, and a vicious cycle is created. Mitochondria damage and ROS production induced by MPTP may impair the protein degradation system and accelerate the toxic aggregation of **α**-syn. Moreover, **α**-syn has also been reported a prion-like spread between cells and the cell-to-cell transmission of pathological **α**-syn may cause more aggregation of **α**-syn and neuronal degeneration [60–63]. The neuronal insults of mitochondria, protein degradation system and oxidative stress, together with the toxic aggregation of **α**-syn may increase the sensitivity of DA neurons to MPTP. And the toxic role of aberrant **α**-syn in neurons as well as the exact mechanisms underlying the interaction with MPTP require further exploration.

Many *in vitro* studies have shown that **α**-syn overexpression enhances cell death following MPP^+^ exposure, and some have reported a resistant effect to MPTP in **α**-syn null mouse models [64]. These studies support the notion that the overexpression of **α**-syn is involved in an increased vulnerability of DA neurons to MPTP. However, Rathke’s research in transgenic mice overexpressing mutant **α**-syn A30P has failed to show heightened susceptibility to MPTP [65]. The motor phenotype and pathological impairments are different between transgenic and rAAV generated **α**-syn mouse models in many studies. It is probable that adaptive changes of DA neurons in response to **α**-syn overexpression prevent heightened sensitivity to MPTP in transgenic models. However, mice generated by rAAV mediated overexpression of the **α**-syn A53T mutant have also failed to show increased sensitivity to MPTP [66]. The overexpression of the **α**-syn A53T mutant did not produce loss of DA neuron and striatal fiber before MPTP treatment in their study, while modest impairments appeared in our **α**-syn overexpressing mice. It’s possible that the expression levels and pathological stages induced by **α**-syn influence the vulnerability to MPTP. Due to the variable effects caused by the overexpression of **α**-syn, the characteristics of neurons with increased sensitivity to MPTP require further clarification.

In conclusion, we used rAAV2/1- **α**-syn vectors to generate progressive nigrostriatal pathology and motor behavior deficits, the lesions mimicked primary features observed in PD patients. Furthermore, we also investigated the influence of neurotoxin MPTP after rAAV vectors mediated overexpression of **α**-syn, and the results suggested increased vulnerability of mice to MPTP. These models extend the role of overexpressed **α**-syn in the nigrostriatal system under conditions of environmental insults, and simultaneously provide effective approaches for exploring the pathologic mechanisms of PD and the development of related therapies.

## Supporting Information

S1 Fig Overexpression of α-syn in the SNpc at 4 weeks after rAAV- α-syn transduction. TH positive staining (Red) and co-staining of human- α-syn (Green) were observed in DA neurons (D-F). The overexpression of GFP was also detected in TH-positive neurons in the SNpc after rAAV2/1-GFP injection (A-C). Western blotting showed significant increases of α-syn in midbrain of rAAV2/1- α-syn injected mice at 4 weeks after transduction (G-H). Data are expressed as mean±SEM of 3 mice. *P<0.05 (unpaired, two-tailed Student’s t test). Scale bar: 100μm.
(TIF)
