# Supplementary figures and images for: Targeted Overexpression of α-Synuclein by rAAV2/1 Vectors Induces Progressive Nigrostriatal Degeneration and Increases Vulnerability to MPTP in Mouse
Source: PLoS One. 2015 Jun 26;10(6):e0131281. doi: 10.1371/journal.pone.0131281 (PMC4483255; doi:10.1371/journal.pone.0131281)

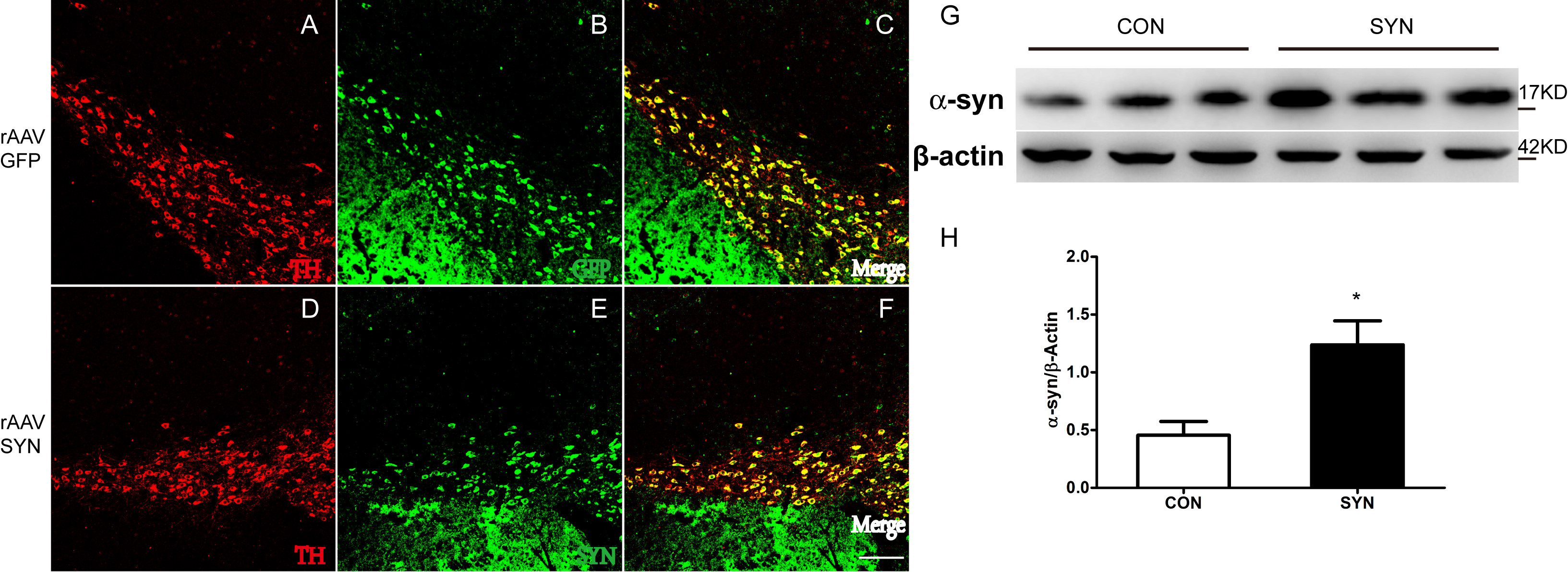

Supplement: S1 Fig — TH positive staining (Red) and co-staining of human- α-syn (Green) were observed in DA neurons (D-F). The overexpression of GFP was also detected in TH-positive neurons in the SNpc after rAAV2/1-GFP injection (A-C). Western blotting showed significant increases of α-syn in midbrain of rAAV2/1- α-syn injected mice at 4 weeks after transduction (G-H). Data are expressed as mean±SEM of 3 mice. *P<0.05 (unpaired, two-tailed Student’s t test). Scale bar: 100μm. (TIF) [file pone.0131281.s001.tif]
